# Chemoradiation impairs myofiber hypertrophic growth in a pediatric tumor model

**DOI:** 10.1038/s41598-020-75913-w

**Published:** 2020-11-11

**Authors:** Nicole D. Paris, Jacob G. Kallenbach, John F. Bachman, Roméo S. Blanc, Carl J. Johnston, Eric Hernady, Jacqueline P. Williams, Joe V. Chakkalakal

**Affiliations:** 1 Department of Pharmacology and Physiology, University of Rochester Medical Center, Rochester, NY USA; 2 Wilmot Cancer Institute, University of Rochester Medical Center, Rochester, NY USA; 3 Stem Cell and Regenerative Medicine Institute, and The Rochester Aging Research Center, University of Rochester Medical Center, Rochester, NY USA; 4 Department of Biomedical Engineering, University of Rochester Medical Center, Rochester, NY USA; 5 Department of Pathology and Laboratory Medicine, Cell Biology of Disease Graduate Program, University of Rochester Medical Center, Rochester, NY USA; 6 Department of Pediatrics, University of Rochester Medical Center, Rochester, NY USA; 7 Department of Radiation Oncology, University of Rochester Medical Center, Rochester, NY USA; 8 Department of Environmental Medicine, University of Rochester Medical Center, Rochester, NY USA; 9 Center for Musculoskeletal Research, University of Rochester Medical Center, Rochester, NY USA

**Keywords:** Cancer models, Cancer therapy, Paediatric cancer, Sarcoma, Cancer, Developmental biology, Ageing, Cell growth, Disease model, Stem cells, Stem cells, Adult stem cells, Ageing, Muscle stem cells

## Abstract

Pediatric cancer treatment often involves chemotherapy and radiation, where off-target effects can include skeletal muscle decline. The effect of such treatments on juvenile skeletal muscle growth has yet to be investigated. We employed a small animal irradiator to administer fractionated hindlimb irradiation to juvenile mice bearing implanted rhabdomyosarcoma (RMS) tumors. Hindlimb-targeted irradiation (3 × 8.2 Gy) of 4-week-old mice successfully eliminated RMS tumors implanted one week prior. After establishment of this preclinical model, a cohort of tumor-bearing mice were injected with the chemotherapeutic drug, vincristine, alone or in combination with fractionated irradiation (5 × 4.8 Gy). Single myofiber analysis of fast-contracting extensor digitorum longus (EDL) and slow-contracting soleus (SOL) muscles was conducted 3 weeks post-treatment. Although a reduction in myofiber size was apparent, EDL and SOL myonuclear number were differentially affected by juvenile irradiation and/or vincristine treatment. In contrast, a decrease in myonuclear domain (myofiber volume/myonucleus) was observed regardless of muscle or treatment. Thus, inhibition of myofiber hypertrophic growth is a consistent feature of pediatric cancer treatment.

## Introduction

One of the great achievements in medicine has been an improved survival rate following pediatric cancer diagnosis, frequently the result of treatments involving combined modalities, such as chemoradiation^1,2^. However, in conjunction with the increased survival rate, many of these individuals have experienced premature indices of physical limitation normally associated with the elderly population^1,3,4^. Importantly, the various direct and indirect effects of interventions such as radiation and chemotherapy, which are utilized in tumor treatment (i.e. killing actively cycling cells, permanently impairing differentiated cell populations through DNA damage, and influencing critical biological processes such as transcription, translation, and microtubule stabilization), also negatively impact growth and maintenance of non-tumor bearing (normal) tissues^5–14^. Thus, we see that prior to the age of 40, many childhood cancer survivors present with signs of skeletal muscle decline associated with sarcopenia, a syndrome of skeletal muscle decline commonly observed in the elderly^15–17^. For example, in a study of acute lymphoblastic leukemia (ALL) survivors whose treatment included combinations of radiation or chemotherapy, participants were reported to have higher body mass index (BMI) values when compared to siblings in adulthood, indicative of skeletal muscle loss^18,19^. Indeed, decline in lower extremity strength is a common long-term finding in ALL survivors^20,21^. Survivors of sarcomas such as rhabdomyosarcoma (RMS), for which the standard of care often includes radiation and chemotherapy, also exhibit late effect musculoskeletal abnormalities and skeletal muscle atrophy^22–24^.

In preclinical models, radiation and drug treatments can induce skeletal muscle decline. For instance, hind limb irradiation of mouse pups with a single fraction of 18 Gy of ionizing radiation leads to skeletal muscle atrophy; however, this phenomenon was not observed after similar irradiation of adult hind limbs^8^. In contrast, atrophy of skeletal muscle fiber (myofiber) subtypes was observed after a 16 Gy single dose to adult lower limbs, but no subtype myofiber atrophy was observed after fractionated doses of 4 × 4 Gy^9^. Furthermore, myofiber atrophy occurs in consequence to treatment of adult mice with a variety of chemotherapeutics, such as doxorubicin, carboplatin, or cisplatin^25–28^. Although radiation or chemotherapeutic treatment can adversely affect skeletal muscle, the basis of radiation or chemotherapy-induced muscular deficits and the consequences of combined treatments, particularly during active stages of skeletal muscle growth and maturation, are unknown.

Prepubertal growth is a period of juvenile development after weaning and prior to the onset of puberty, with the induction of sex hormones marking the beginning of adolescence^29–32^. As prepubertal growth proceeds, skeletal muscles undergo extensive growth and maturation^30^. Skeletal muscle is composed of a heterogeneous mixture of long multinucleated cells, or myofibers, that function as primary effectors for force production. During juvenile growth, a robust increase in the cross-sectional area (CSA) of myofibers occurs^30^. This increase in myofiber CSA reflects both myonuclear accretion and myonuclear domain expansion^30^. Myonuclear accretion refers to the addition of new myonuclei, whereas myonuclear domain is a measure of the volume of sarcoplasm supported by an individual myonucleus^30,33–35^. Although the prepubertal period is a time of extensive skeletal muscle growth, the potential consequences of childhood stressors, such as radiotherapy or chemotherapy, on myofiber maturation have not been investigated. Furthermore, the effects of combined radiation and chemotherapeutic drug administration on skeletal muscle in the context of tumor treatment have yet to be examined. Finally, whether juvenile stressors that negatively impact myofiber growth manifest in a reduction of myonuclei, domain, or both is unknown.

Here, we report on the development and characterization of a preclinical mouse model of pediatric RMS therapy. Initially, using intravital imaging, we characterized the growth of RMS tumors from a previously described C57BL/6 syngeneic RMS cell line in mouse hind limbs. We found that RMS tumors are amenable to treatment with local fractionated X-radiation conducted with a Small Animal Radiation Research Platform (SARRP). Furthermore, the combination of a fractionated localized administration of radiation with systemic treatment of the chemotherapeutic vinca alkaloid, vincristine, was efficient at achieving RMS tumor elimination, while vincristine alone was a less effective treatment. Subsequently, we examined the radiation-induced and off-target effects of RMS treatment in tumor-bearing prepubertal mice utilizing localized fractionated radiation with or without vincristine. We determined that fractionated radiation and/or vincristine treatment, administered during the juvenile period, leads to a reduction in adult myofiber size. Although pediatric cancer treatment resulted in reduced adult myofiber size, loss of myonuclei depended on muscle examined and therapeutic regimen. Whereas a reduction of myonuclei varied, a decrease in adult myonuclear domain consistent with inhibition of hypertrophic growth was observed regardless of juvenile treatment strategy or skeletal muscle examined.

## Results

### Characterization of RMS cell orthotopic implantation

**Figure 1:**
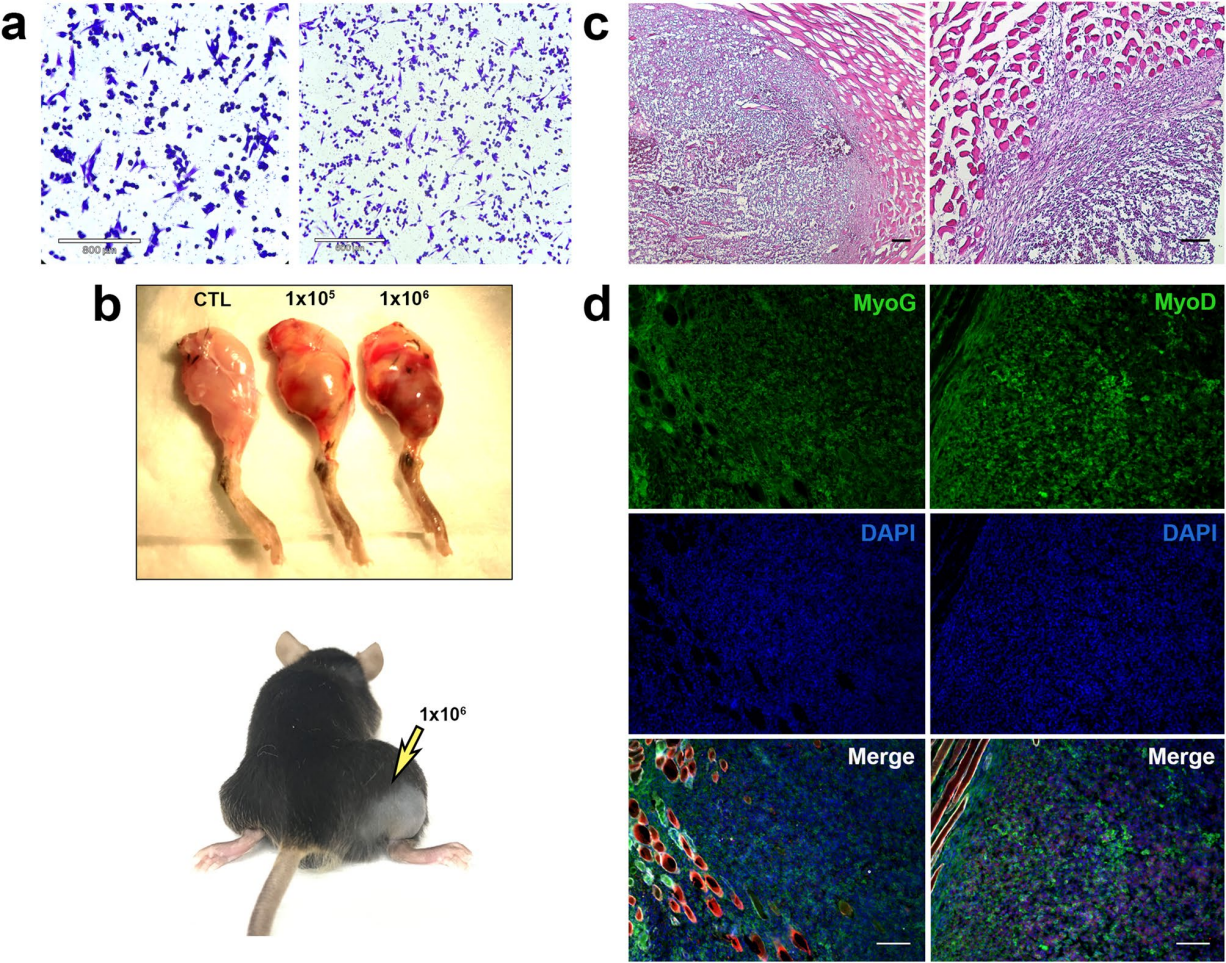
Characterization of implanted rhabdomyosarcoma tumors from cell line M3-9-M. (**a**) Representative brightfield images of crystal violet-stained rhabdomyosarcoma (RMS) cells grown for 3 days in culture. Scale 800 µm (left), 600 µm (right). (**b**) Representative photos of C57BL/6 hindlimbs (top) and mouse (bottom) 1 week following implantation of RMS cells at varying concentrations. (**c**) RMS tumor morphology by H&E staining of longitudinal sections (left) and cross-sections (right). Scale 100 µm. (**d**) Representative images of immunostaining of tumor sections to detect muscle-progenitor proteins Myogenin or MyoD (green), DAPI (blue), Laminin (white), and Myosin (red). Scale 100 µm.

To examine the consequences of irradiation and/or chemotherapeutic administration on juvenile skeletal muscle integrity, we first sought to establish a pediatric tumor model during the prepubertal period. For this purpose, we focused on a relatively prevalent childhood cancer, rhabdomyosarcoma (RMS). RMS primary tumors are composed of malignant undifferentiated muscle cells that express myogenic progenitor markers^36,37^. We obtained and evaluated a previously established C57BL/6 syngeneic M3-9-M RMS cell line. M3-9-M cells were isolated from spontaneous tumors in mice heterozygous for transgenic overexpression of hepatocyte growth factor and loss of p53^36^. When cultured, these adherent RMS cells maintain a round or spindle-shaped appearance typical of immature myoblasts (Fig. 1a). To establish our model, M3-9-M RMS cells were resuspended in sterile PBS and injected intramuscularly into the right gastrocnemius muscle of young (3–4-week-old) C57BL/6 mice at previously described concentrations with the least likelihood to result in metastases^36^. One week following implantation, rhabdomyosarcoma tumors were large (~ 20 mm^3^) and vascularized, filling the entire gastrocnemius and interfering with normal ambulation (Fig. 1b). Upon dissection of these muscles, the tumor was shown to be in situ, contained within the gastrocnemius fascia, although the soleus and plantaris muscles were displaced and visibly smaller than those from the contralateral side. Cryosectioning of fixed gastrocnemius muscles harvested at this time revealed small, densely packed cells positive for Myogenin and MyoD surrounded by normal muscle fibers (Fig. 1c,d).

### Intravital imaging to track RMS tumor progression

**Figure 2:**
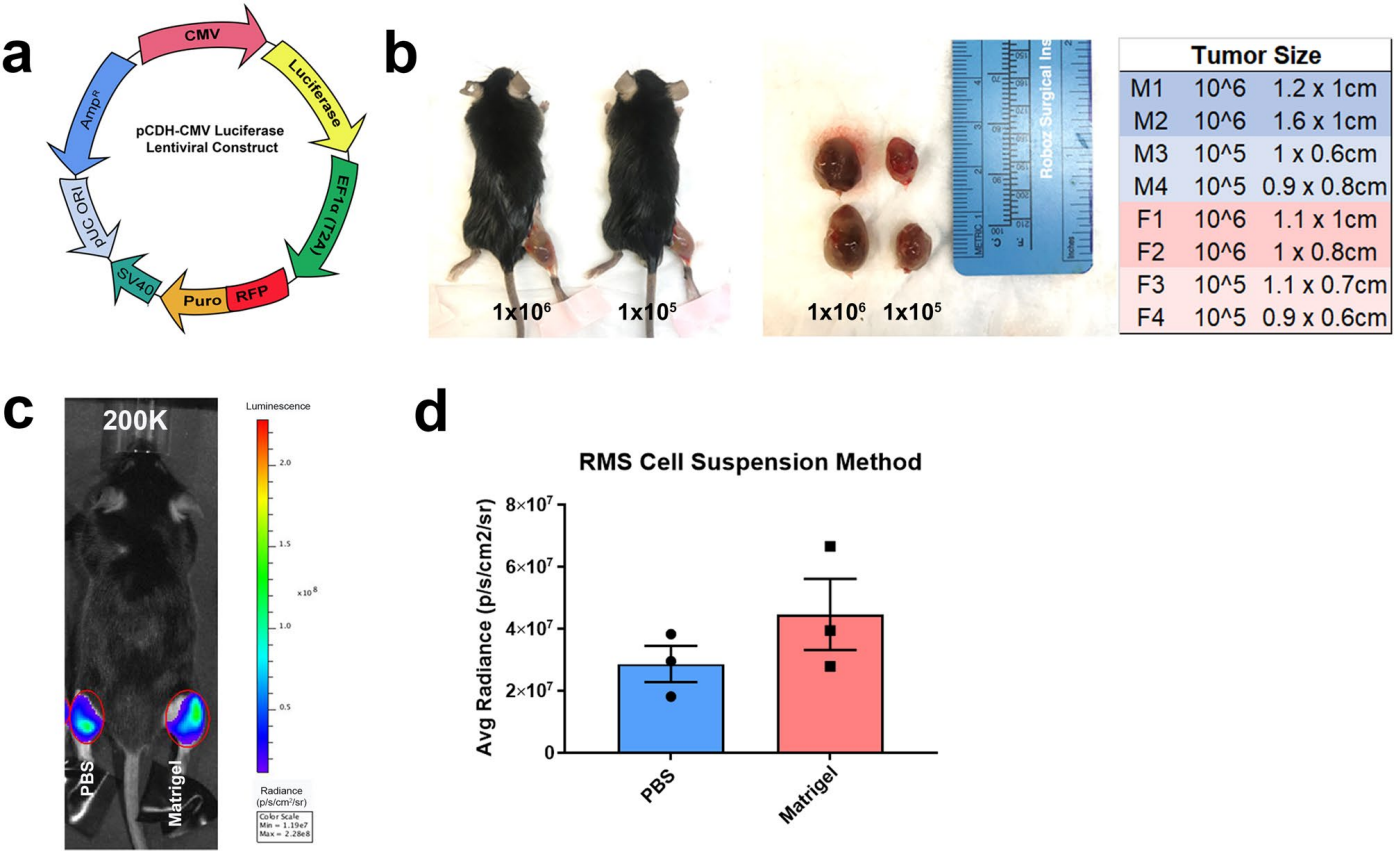
Establishment of rhabdomyosarcoma cell line RMS-luc-RFP as a cancer model for hindlimb radiotherapy. (**a**) Schematic depicting the luciferase-RFP construct stably expressed by RMS-luc-RFP cells. (**b**) Representative photos of C57BL/6 mice (left) and tumor-bearing gastrocnemius muscles (right) 1 week following implantation of RMS-luc-RFP cells at varying concentrations, with tumor sizes. (**c**) Representative brightfield/luminescent image and (**d**) quantification of luminescence by IVIS of RMS-luc-RFP cells implanted at varying concentrations (100–300 K) in PBS with or without matrigel present (1:1 PBS/Matrigel), N = 3 mice.

To enable longitudinal analysis of tumor growth/recession with treatment, it was necessary that we incorporate a reporter system into the M3-9-M RMS cell line. The luciferase gene was inserted into a commercially available pCDH-CMV-RFP lentiviral vector, which was used to stably infect RMS cells with a luciferase-RFP reporter and establish the cell line we hereafter refer to as RMS-luc-RFP (Fig. 2a). We then confirmed that incorporation of the lentiviral vector did not impair the aggressive growth of RMS cells (Fig. 2b). The expression of luciferase enabled us to utilize the intravital imaging capabilities of the In Vivo Imaging System (IVIS) to track RMS-luc-RFP cells following injection. With the ability to more accurately determine RMS tumor size and density, we compared the efficacy of different cell implantation methods. We then tested whether tumors would grow more robustly and/or locally if cells were resuspended in a sterile PBS/Matrigel (1:1) mixture prior to implantation rather than in PBS alone. IVIS analysis one-week post-implantation of 100,000–300,000 cells revealed a trend of increased luminescence of RMS tumors implanted with Matrigel (Fig. 2c,d). These findings established the narrow time window in which to treat the primary RMS tumor in this model.

### Irradiation of RMS tumors

**Figure 3:**
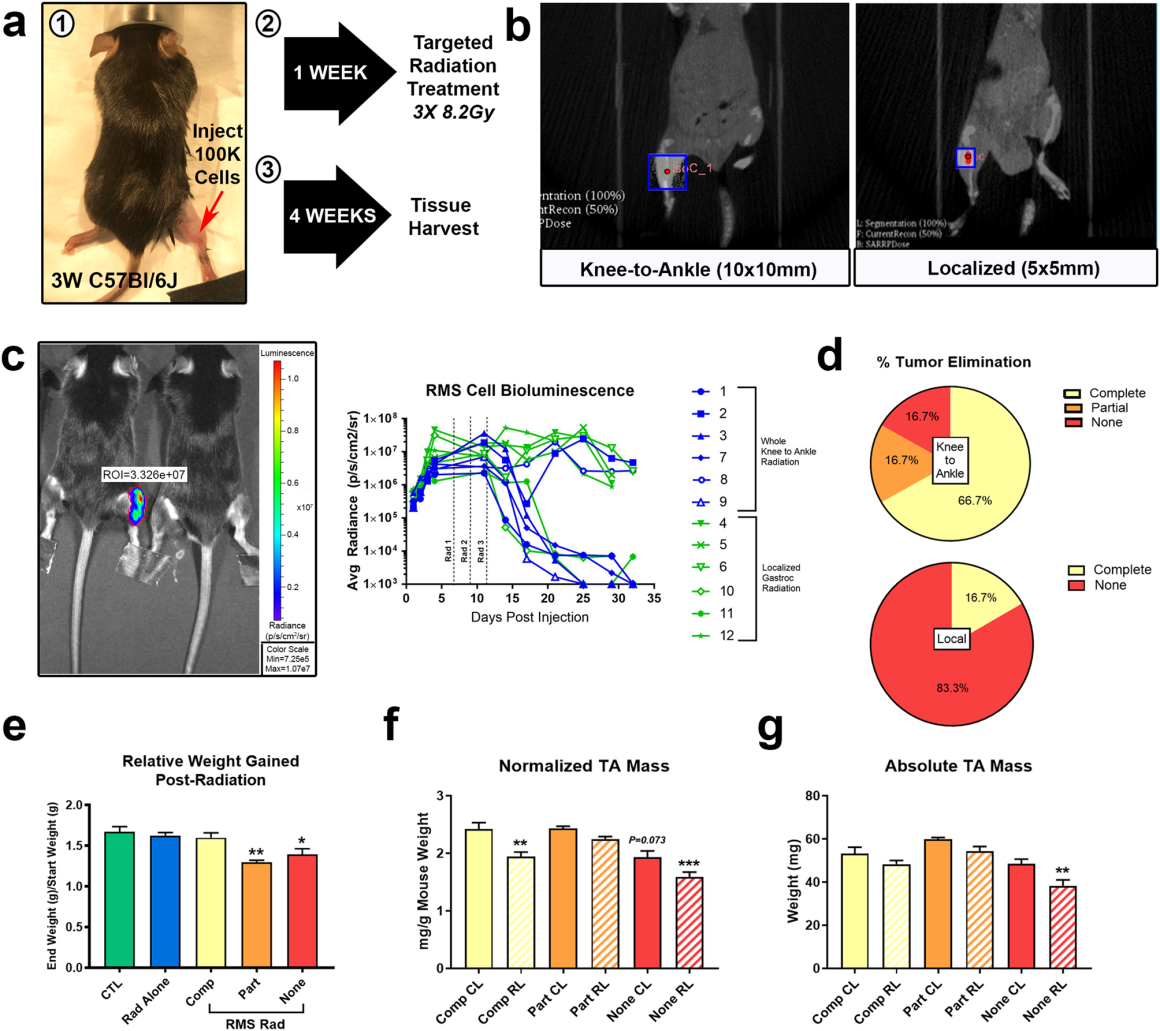
Targeted hindlimb irradiation during juvenile stages effectively eliminates implanted rhabdomyosarcoma tumors. (**a**) Schematic describing the method of RMS tumor cell implant into right gastrocnemius, subsequent radiation treatment, and tissue harvest. (**b**) Representative CT images of two methods of hindlimb targeted radiotherapy, knee-to-ankle (left) and localized to relative tumor location (right). Boxes represent radiation field; red circles label the isocenter. (**c**) IVIS luminescent detection merged with brightfield image (left) and quantified as average radiance (p/s/cm^2^/sr) recorded biweekly for 5 weeks post tumor cell implantation (right), n = 6 mice. (**d**) Quantification of percentages of tumor elimination classified as complete (no tumor), partial (tumor < 12 mm), or none (tumor persisted > 12 mm) based on gross measurement at time of harvest, n = 6 mice/group. (**e**) Ratio of weight at time of harvest to weight at four weeks old was quantified. Tibialis anterior (TA) mass shown as (**f**) normalized to mouse body weight or (**g**) absolute mass, n = 3 mice/group. *P < 0.05, **P < 0.01, ***P < 0.001, to CTL or Comp CL. One-way ANOVA, Tukey’s test. *Comp* complete, *Part* partial, *CL* contralateral leg, *RL* irradiated leg.

To test irradiation as an intervention in our pediatric tumor model, we used a clinically relevant method of radiation delivery, the Small Animal Radiation Research Platform (SARRP). Following RMS cell inoculation of 3-week-old C57Bl/6 J mice, tumors were left to grow for approximately 1 week prior to the start of irradiation and tissue was harvested three weeks post-radiation (Fig. 3a). Tumors were treated with either knee-to-ankle (10 × 10 mm field) or localized (5 × 5 mm field) fractionated x-ray irradiation (3X 8.2 Gy, M,W,F) with the SARRP (Fig. 3b). Of note, standard clinical regimens for individuals afflicted with high grade RMS utilize ~ 2 Gy fractions for total doses of 35–50 Gy^38–40^. However, in response to the availability constraints of the SARRP and to fit the fractionation schedule within the prepubertal period of mouse muscle growth (4–6 weeks old)^30^, we used a hypo-fractionated schedule with a biological effective dose (BED) equivalent to a total radiation dose at the lower end of the clinical regimen range, using an α/ß ratio of 3. Additionally, we confirmed that a single dose of 8.2 Gy was insufficient to consistently eliminate tumors (Supplementary Fig. S1). Using the fractionated regimen, with weekly IVIS screening, we observed an ~ 70% successful tumor regression rate using the knee-to-ankle protocol. In contrast, only a ~ 20% regression rate was seen using the smaller field, which encompassed roughly the site of initial implantation (Fig. 3c,d). Notably, intravital imaging also confirmed significant RMS tumor growth during the period prior to the first dose of radiation, establishing a relevant tumor size pre-treatment. Furthermore, we assessed the overall change in mouse body weight from four to eight weeks as well as the tibialis anterior (TA) muscle mass at time of harvest, which revealed that the only systemic changes indicative of a cancer cachexia-like phenotype were seen in the mice with partial or full tumor recurrence (Fig. 3e–g).

Through multiple subsequent experimental cohorts, we established that use of a 10 × 10 mm irradiation field consistently resulted in tumor ablation; the field was defined distally by the transition of the gastrocnemius to the Achilles tendon, with the proximal border falling just above the knee joint (Supplementary Fig. S2a). The landmarks were necessary given that RMS tumors are soft tissue sarcomas and are not visually distinguishable from normal tissue in a CT image. Furthermore, use of a consistent field size enabled reproducibility between individual animals; use of the SARRP’s on-board CT assisted with the consistent and repetitive placement of the radiation field through correct orientation of the right leg in the SARRP bed (Supplementary Fig. S2b).

### Dual therapy juvenile cancer treatment model

**Figure 4:**
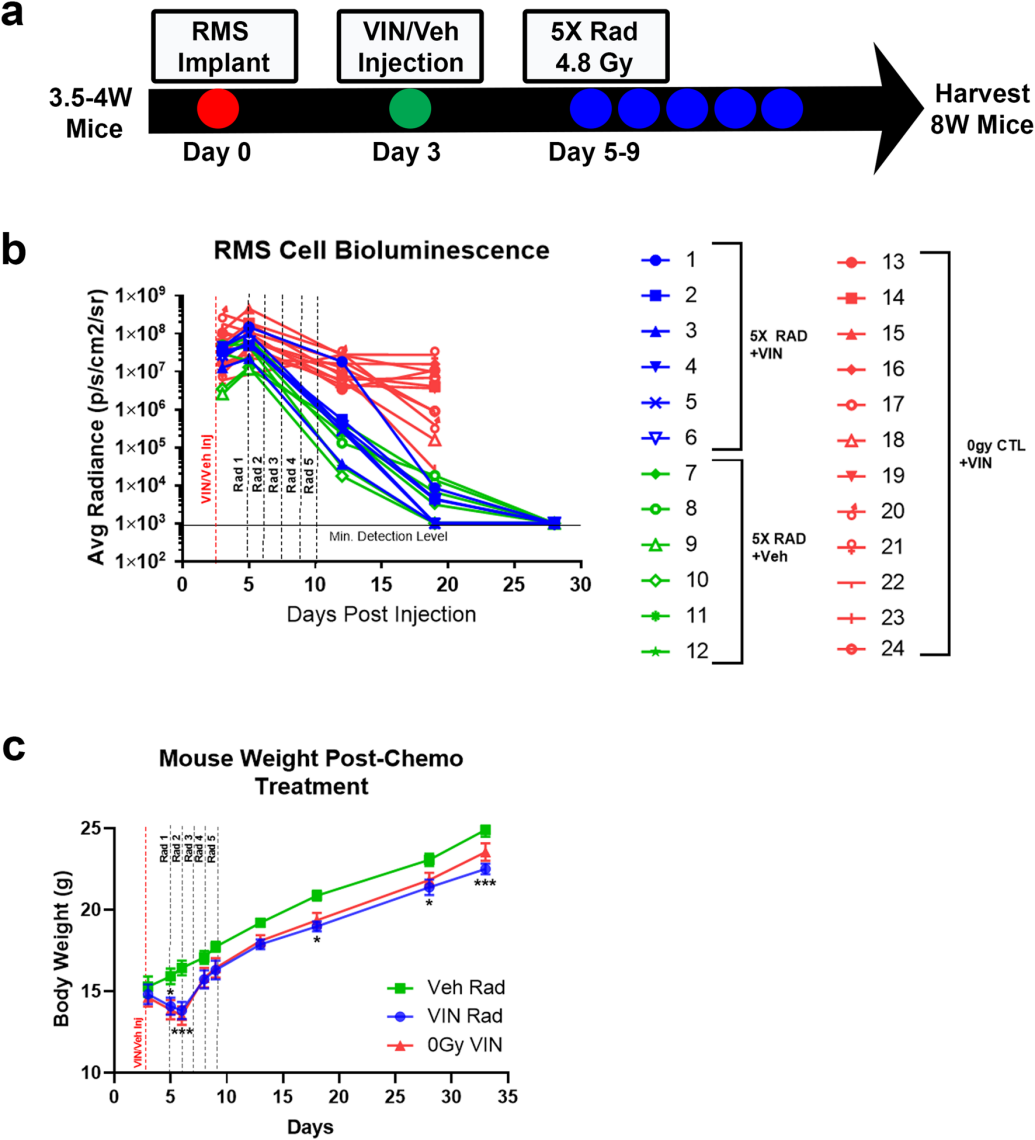
Juvenile hindlimb irradiation in conjunction with chemotherapy treatment effectively eliminates RMS tumors and is a pre-clinical mouse model of pediatric RMS. (**a**) Schematic describing the timeline of RMS tumor cell implant into 3.5–4-week-old mice, Vincristine (VIN) or vehicle treatment, subsequent radiation treatment, and tissue harvest at 8 weeks old. (**b**) IVIS bioluminescent detection quantified as average radiance (p/s/cm^2^/sr) recorded for 5 weeks post tumor cell implantation. (**c**) Mouse body weight at time of VIN or vehicle injection until time of harvest was quantified. N = 6 mice. *P < 0.05, ***P < 0.001, VIN Rad to Veh Rad, Two-way ANOVA, Tukey’s multiple comparisons test. *CL* contralateral leg, *RL* irradiated leg.

Multimodal chemotherapy, often including the vinca alkaloid, vincristine, in combination with a fractionated radiation regimen is commonly used to treat RMS^41,42^. Therefore, we next tested the ability of vincristine to treat RMS tumors alone or in combination with a hypo-fractionated radiation schedule (5X 4.8 Gy M-F) during juvenile mouse growth. The revised radiation schedule again had a BED equivalent to a total radiation dose at the lower end of the clinical range, but now used an α/ß ratio of 10 to account for the observed acute response. Following implantation of RMS-luc-RFP cells into the right gastrocnemius muscle of 3.5–4-week-old mice (Day 0), a single IP injection of vehicle (saline) or vincristine was administered (Day 3). Subsequently, cohorts of tumor-bearing mice were treated with (Rad) or without (0 Gy CTL) fractionated radiation (Day 5–9), with tissue harvest occurring three weeks post-irradiation (Fig. 4a). IVIS analysis and gross examination revealed that vincristine alone did not lead to complete tumor ablation, although this treatment led to a partial reduction in RMS tumor size, albeit inconsistently (Fig. 4b) (Supplementary Figure S3). As expected, vincristine treatment with fractionated irradiation was associated with complete elimination of tumors; however, there was a substantial loss in body weight in the days immediately following drug administration, which failed to return to baseline over the observation period (Fig. 4c).

### Prepubertal radiation and vincristine administration inhibit myofiber hypertrophic growth

**Figure 5:**
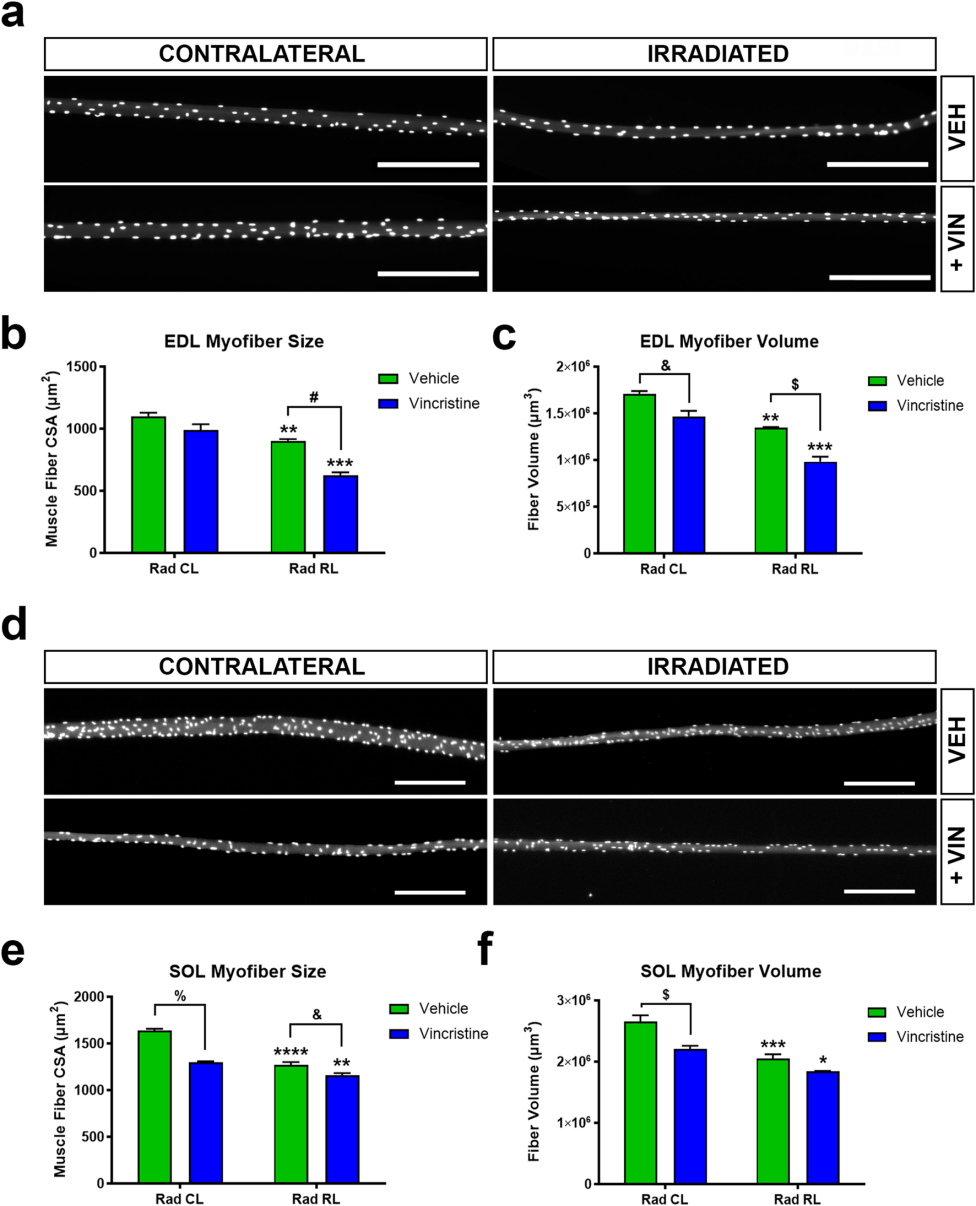
Juvenile irradiated mice experience muscle atrophy four weeks following treatment which is exacerbated by chemotherapy. (**a**) Representative images of contralateral and irradiated, vehicle or Vincristine treated DAPI-stained fixed EDL single fibers, Scale = 200 μm. (**b**) EDL myofiber average cross-sectional area (CSA). (**c**) EDL myofiber volume (CSA × Length). (**d**) Representative images of contralateral and irradiated, vehicle or Vincristine treated DAPI-stained fixed Soleus (SOL) single fibers, Scale = 200 μm. (**e**) SOL myofiber average cross-sectional area (CSA). (**f**) SOL myofiber volume (CSA × Length). **P < 0.01, ***P < 0.001, to CL. ^&^P < 0.05, ^$^P < 0.01, ^#^P < 0.001, ^%^P < 0.0001 to Vehicle CL or RL. Two-way ANOVA, Tukey’s multiple comparisons test. *CL* contralateral leg, *RL* irradiated leg. 50 myofibers/muscle, N = 3 mice/group.

During early postnatal and prepubertal development, extensive skeletal muscle hypertrophic growth occurs^30^. To assess the consequences of fractionated radiation and vincristine treatment on hypertrophic growth in this time period, the cross-sectional area (CSA) and volume of fixed lower limb extensor digitorum longus (EDL) and soleus (SOL) muscle myofibers were measured in young adult mice (8 weeks old)^30,43^. The EDL and SOL are representative skeletal muscles composed primarily of faster and slower-contracting myofibers respectively^30^. In comparison to contralateral (CL) EDL myofibers from vehicle treated mice, CL EDL myofibers from vincristine treated mice displayed a 10% (1100 µm^2^ vs 990 µm^2^) and 15% (1.71 × 10^6^ µm^3^ vs 1.47 × 10^6^  µm^3^) reduction in CSA and volume respectively. When comparing vehicle treated CL vs irradiated (RL) EDL muscles, RL myofibers displayed slightly more atrophy with a 18% (1100 µm^2^ vs 904 µm^2^) and 22% (1.71 × 10^6^  µm^3^ vs 1.35 × 10^6^  µm^3^) reduction in CSA and volume. The greatest extent of EDL myofiber atrophy was observed with combined radiation/vincristine treatment. There was a 43% reduction in both CSA (1100 vs 627 µm^2^) and volume (1.71 × 10^6^  µm^3^ vs 0.98 × 10^6^  µm^3^) of vincristine treated RL EDL myofibers relative to vehicle CL EDL myofibers. Strikingly, vincristine treated RL myofibers displayed a more substantially reduced CSA (30%) and volume (27%) than seen with radiation alone, indicating an additive effect on the inhibition of EDL myofiber hypertrophic growth (Fig. 5a–c).

Similarly, prepubertal radiation and vincristine treatment also inhibited the hypertrophic growth of SOL myofibers. Relative to CL SOL myofibers with vehicle treatment, a 20% (1635 µm^2^ vs 130 µm^2^) and 17% (2.65 × 106  µm^3^ vs 2.20 × 106  µm^3^) reduction in CSA and volume was observed in vincristine treated CL SOL myofibers, revealing a more substantial systemic effect of the drug alone. We observed a 22% (1635 µm^2^ vs 1301 µm^2^) and 23% (2.65 × 106  µm^3^ vs 2.20 × 106  µm^3^) reduction in CSA and volume in vehicle treated CL vs RL SOL myofibers. Also, combined treatments had a modest additive effect on the inhibition of SOL myofiber hypertrophic growth. Specifically, relative to CL SOL myofibers from vehicle treated mice, a 29% (1635 µm^2^ vs 1159 µm^2^) and 31% (2.65 × 106  µm^3^ vs 1.84 × 106  µm^3^) reduction in CSA and volume respectively was observed in RL SOL myofibers from vincristine treated mice (Fig. 5d–f).

### Prepubertal radiation and vincristine administration reduce adult myonuclear number and domain

**Figure 6:**
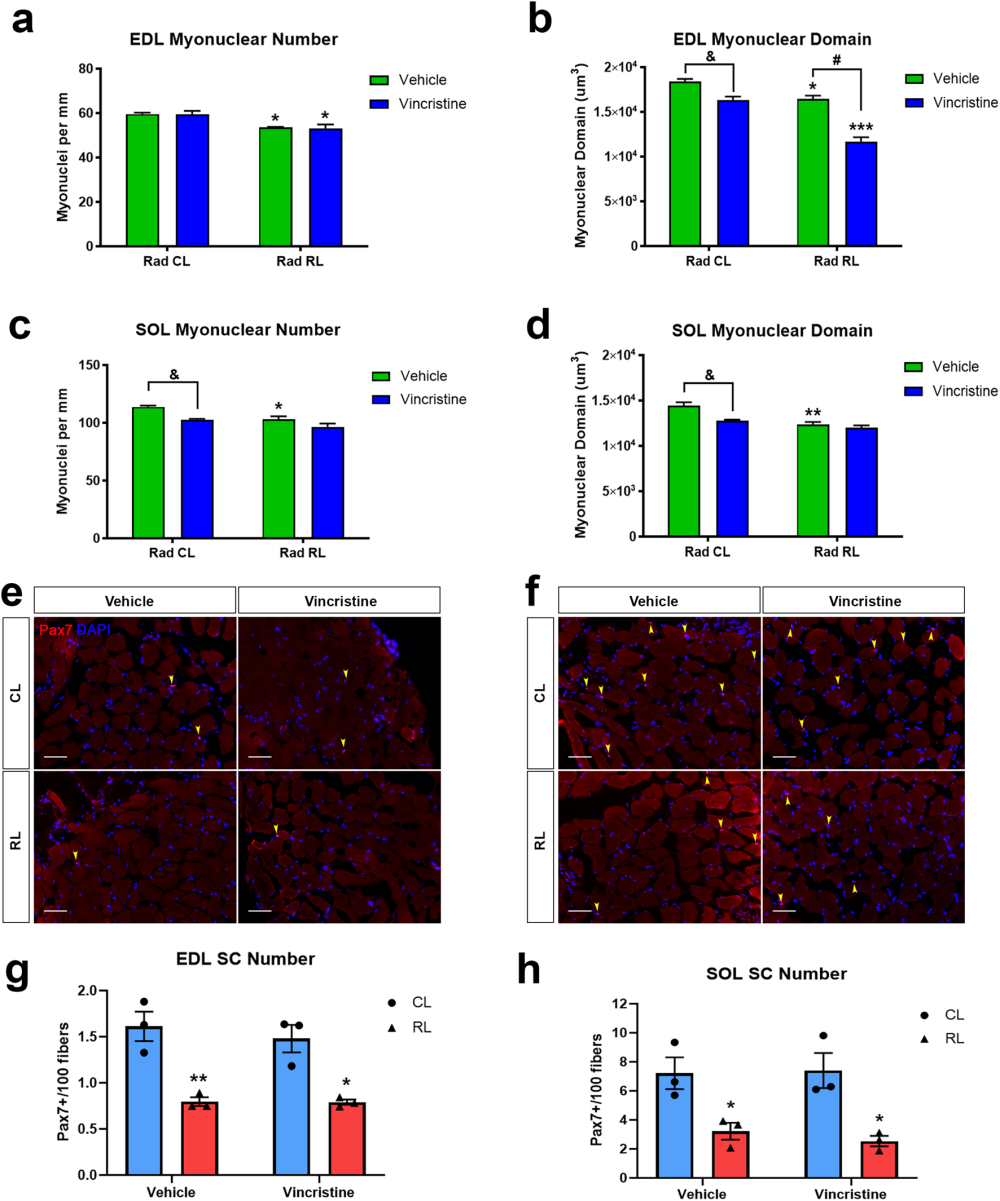
Chemotherapy treatment causes a synergistic decline of EDL myonuclear domain and results in decreased SOL myonuclear number with no effect on SC number. (**a**) EDL myonuclei number per mm of myofiber length. (**b**) EDL myonuclear domain ((CSA × Length)/Total MN). (**c**) SOL myonuclei number per mm of myofiber length. (**d**) SOL myonuclear domain ((CSA × Length)/Total MN). Representative Pax7 (red), DAPI (blue) immunofluorescent images and quantification of cross-sections of (**e**,**g**) EDL and (**f**,**h**) SOL. Scale = 50 µm. *P < 0.05, **P < 0.01, ***P < 0.001, to CL. ^&^P < 0.05, ^#^P < 0.001, to Vehicle CL or RL (**a**–**d**) or to CL of same condition (**g**,**h**). Two-way ANOVA, Tukey’s multiple comparisons test. *CL* contralateral leg, *RL* irradiated leg. N = 3 mice/group.

In rodents and humans, prepubertal myofiber growth is associated with myonuclear accretion^30,44^. To assess the effect of multimodal juvenile cancer therapy on myonuclear number, we counted the number of myonuclei per millimeter (MN/mm) within EDL and SOL myofibers of the treated mice as they entered early adulthood (8 weeks old). In vehicle compared to vincristine treated CL EDL myofibers, we did not observe a loss of myonuclei (60 MN/mm); however, radiation regardless of vehicle or vincristine treatment led to a 10–12% (60 MN/mm vs 54–53 MN/mm) decline in myonuclear number relative to CL EDL myofibers (Fig. 6a). Examination of myonuclear number in CL SOL myofibers revealed a 10% loss in vincristine relative to vehicle treated mice (103 MN/mm vs 114 MN/mm). Irradiation led to a 10 or 15% (114 MN/mm vs 103 or 97 MN/mm) decline in SOL myonuclear number in relation to CL myofibers with vehicle or vincristine treatment, respectively, thus no further deficit occurred due to combined treatment (Fig. 6c).

Expansion of myonuclear domain reflects an increase in the ratio of myofiber cytoplasm (sarcoplasm) to myonuclei^34^. Prepubertal myofiber growth is characterized by a marked increase in myonuclear domain^30^. Thus, we sought to determine the effects of radiation and vincristine treatment on this process during juvenile growth in EDL and SOL muscles. Assessment of myonuclear domain in CL EDL myofibers from vincristine treated mice revealed a 22% decrease relative to vehicle treated CL myofibers (18,396  µm^3^ vs 16,316  µm^3^). Similarly, analysis of vehicle treated CL vs RL EDL myofibers demonstrated a 21% decline in myonuclear domain (18,396 µm^3^ vs 16,476 µm^3^). Remarkably, we observed a further 30% decrease of myonuclear domain in RL EDL myofibers from vincristine treated mice relative to their vehicle treated counterparts (16,476 µm^3^ vs 11,656 µm^3^) (Fig. 6b).

Prepubertal radiation and vincristine treatment was also found to inhibit the expansion of myonuclear domain in SOL myofibers. Relative to vehicle treated CL SOL myofibers, those from vincristine treated mice experienced a 12% reduction in myonuclear domain (14,441 µm^3^ vs 12,774 µm^3^). In comparing vehicle treated CL to RL SOL myofibers, there was a 14% reduction in myonuclear domain (14,441 µm^3^ vs 12,355 µm^3^). We observed a 3% additional reduction in myonuclear domain when RL SOL myofibers were treated with vincristine rather than vehicle (12,355 µm^3^ vs 11,994 µm^3^) (Fig. 6d). Overall, detailed statistical analysis confirmed that there was a cumulative impairment of myofibers in regard to reduced size and myonuclear domain when both chemotherapy and irradiation were delivered, indicating a significant interaction between the two (Supplementary Fig. S4).

Skeletal muscle is endowed with a population of Pax7-expressing resident stem cells called satellite cells (SCs), which are a principal source of myonuclei that contribute to embryonic and early postnatal growth as well as muscle regeneration^30,45–48^. Since we observed a loss of myonuclei, particularly after prepubertal radiation, EDL and SOL muscles were processed for Pax7 immunostaining to assess SC content at 3 weeks post-treatment. In irradiated (RL) muscles we observed an approximately two-fold reduction in SCs in EDL (~ 1.5 vs 0.75 Pax7+ cells per 100 fibers) and SOL (~ 7 vs 3 Pax7+ cells per 100 fibers) muscle sections (Fig. 6e–h). In contrast, we saw no significant loss of SCs when comparing muscles exposed to vincristine only (vehicle vs vincristine CL).

### Mice exposed to chemoradiation have reduced muscular function

**Figure 7:**
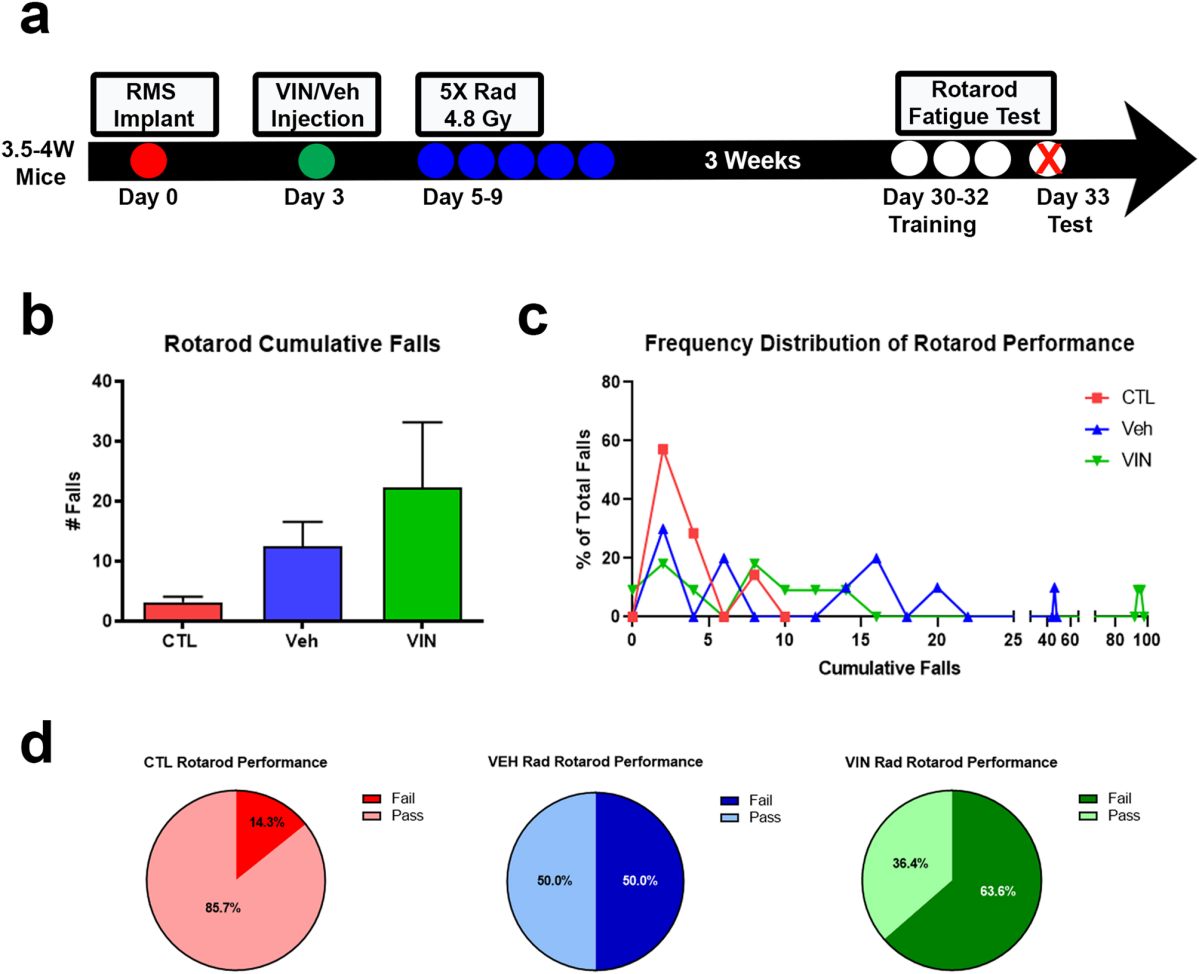
Chemoradiation treatment results in worsened performance in a Rotarod test. (**a**) Schematic describing the timeline of RMS tumor cell implant into 3.5–4-week-old mice, Vincristine (VIN) or vehicle treatment, subsequent radiation treatment, and rotarod training and endurance fatigue test at 8 weeks old. (**b**) Rotarod endurance fatigue test results quantification of average number of cumulative falls over one-hour testing period for each condition. (**c**) Frequency distribution of rotarod endurance fatigue test performance for each condition. (**d**) Quantification of percent of mice in each condition to pass (< 7 falls) or fail (> 7 falls) the rotarod endurance fatigue test. N = 7 mice.

To determine the functional consequences of the myofiber phenotypes we observed, we utilized an established rotarod assay which tests endurance, requiring balance and motor coordination for a prolonged time^49^. At 3 weeks post-chemoradiation, mice underwent three days of training to acclimate to the rotarod prior to the day of testing (Fig. 7a). The test consisted of one hour on the rotarod with increasing speed at 5-min intervals, thus falls per interval were recorded. On average, the vehicle and vincristine irradiated mice experienced roughly ten and twenty times more falls than control mice, respectively (Fig. 7b). When frequency distribution of the number of falls was performed, a rightward shift, or increase in falls, was clearly present in the vehicle cohort and exacerbated with vincristine treatment (Fig. 7c). Subsequently, we set a cut-off of 7 falls as an indicator of “pass” or “fail” status. Based on average control test results, vehicle and vincristine mice have an increasingly higher percentage of test failure (14.3% vs 50% vs 63.6%) (Fig. 7d). Due to the similarities in results, male and female mice were pooled for this experiment.

Reduced muscular function demonstrated in the rotarod test could result from intrinsic myofiber adaptations. Most skeletal muscles possess a mixture of Myosin Heavy Chain (MyHC) isoforms (type I, IIA, IIX, and IIB) that regulate different contraction speeds^50^. We investigated the possibility of myofiber type shifts in EDL or SOL muscles, since this phenotype is observed in neuromuscular diseases or denervation^51,52^. Assessment of immunofluorescent staining for MyHC isoforms in EDL and SOL muscle sections revealed no significant differences in myofiber type due to irradiation or vincristine treatment (Supplementary Fig. S5).

## Discussion

Myonuclear accretion during juvenile muscle development continues up to at least 6 weeks of age (puberty onset) in the mouse, and early adolescence in humans^30,44,53–55^. The adult quiescent SC pool is not fully established until young adult age, and contribution of active SCs is necessary for prepubertal myofiber growth^30,56^. Prepubertal SC ablation leaves both the adult EDL and SOL muscles with reduced myofiber size and myonuclear number^30^. Similarly, we find that prepubertal irradiation is able to reduce adult myofiber size and myonuclear content in these muscles. This supports the disruption of SCs through methods that directly target active cells as a source of myonuclear content deficit. In fact, high doses of γ radiation to proliferating adult SC cultures result in loss of roughly 70% of cells partly due to increased apoptosis and the inhibition of cell cycle progression^57^. Indeed, we observed a 50% of reduction of SCs in EDL and SOL muscles after prepubertal radiation.

Mechanistically, vincristine acts as a microtubule destabilizer and axonal transport blocker. It is also unavoidable that it be administered systemically, potentially affecting the entire body. Vincristine causes mitotic catastrophe at metaphase that induces cell death programs in cancerous and rapidly dividing cells^58–60^. Surprisingly, a single injection of vincristine at 4–5 weeks of age induced loss of myonuclei in SOL as opposed to EDL muscles; however, this was not accompanied by a loss of SCs. Thus, the loss of myonuclei in SOL myofibers after prepubertal vincristine treatment likely involves mechanisms that regulate active SC function and/or myogenic progenitor differentiation^45,61,62^. Vincristine has also previously been shown to impair physiological skeletal muscle function in rats in a dose dependent manner, and its known toxicity profile manifests as neuropathy and muscle atrophy in humans^20,63,64^. Based on the rotarod assay, we observed more pronounced deficits in motor coordination and neuromuscular function in prepubertal radiated and vincristine treated muscles. Alterations in myofiber type proportions are a hallmark feature of major neuromuscular and motor unit rearrangement or loss^51,52^. Although our assessment of myofiber type after radiation or chemotherapy did not reveal any significant modifications, these findings do not rule out other subtler neuromuscular impairments. For instance, the post-synaptic region of the neuromuscular junction (NMJ) is comprised of an enriched microtubule network and specialized sub-synaptic myonuclei^52,65–71^. Furthermore, microtubule destabilization regulates motor axon branch loss during postnatal development^72^. Considering that a single injection of vincristine led to atrophy and a reduction of myonuclear domain in both EDL and SOL muscles, it will be of interest to determine the effects of vincristine on NMJ integrity^52,68,73^. Additionally, the consistent reduction in myonuclear domain seen with vincristine suggests that modulation of signals involved in skeletal muscle hypertrophy and/or atrophy likely have principal roles in vincristine-mediated myofiber adaptations^74^.

Pediatric patients are uniquely vulnerable to the effects of radiotherapy due to the greater number of dividing cells relative to adults. As a general principle, fractionated radiation is employed clinically as a means of minimizing off-target damage, based on the assumption that normal tissues have more effective damage repair apparatus than tumors, a strategy which appears well tolerated by pediatric patients^38,75^. When choosing an irradiation schedule, consideration must be given to fraction size as well as timing between fractions, balancing normal tissue damage against the goal of eliminating tumors^76^. A small animal irradiation system, such as the SARRP, provides a novel opportunity to develop and assess relevant preclinical models^77^. This is, in part, due to the ability to reproducibly perform image-guided (CT) local administration of clinically relevant fractionated radiation schedules, features that are not generally available in many rodent models of radiotherapy^78^. Furthermore, in terms of our focus on modeling normal tissue damage as a consequence of multimodal cancer therapy, our study is the only one to have been performed in an orthotopic immunocompetent tumor model. Here, using the SARRP, we have developed a pediatric tumor model that makes use of a fractionated radiation schedule, titrated to result in successful tumor elimination, and that can be combined with a chemotherapeutic agent during the relatively restricted mouse prepubertal growth period.

**Figure 8:**
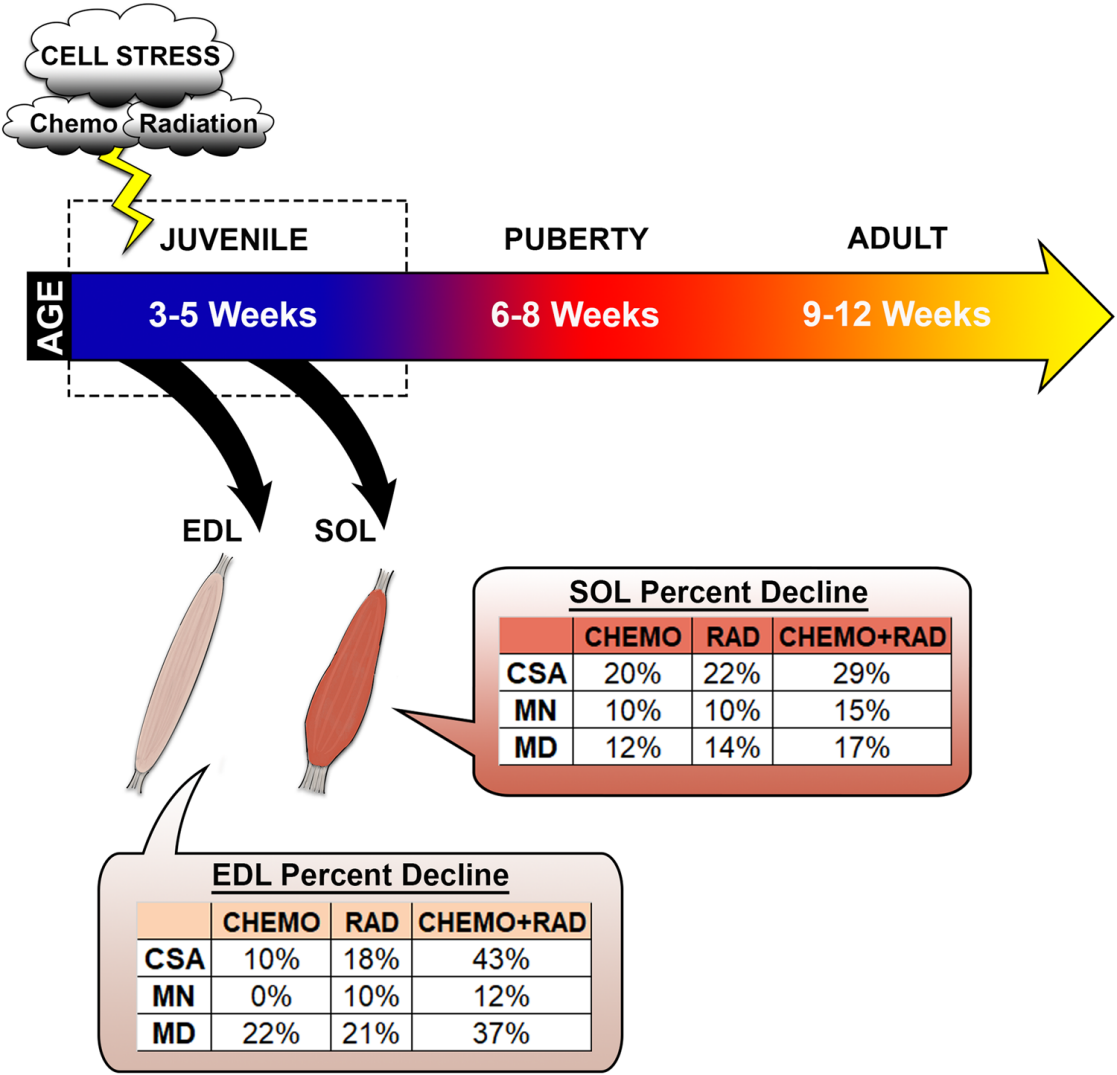
Working model of muscle-specific late effects of juvenile cancer therapies. Schematic demonstrating the different phenotypes associated with either EDL or SOL muscles when chemotherapy, targeted radiation, or both are administered to mice during the juvenile growth period. *CSA* cross-sectional area, *MN* myonuclear number, *MD* myonuclear domain.

Here we find that the combination of juvenile irradiation and a single administration of the chemotherapeutic agent, vincristine, have both distinct individual and synergistic long-term negative effects on adult muscle integrity. A comparison of the data, summarized by the mean percent decrease of myofiber size, myonuclear number, and myonuclear domain, indicates that the inhibition of myofiber hypertrophic growth after irradiation and/or vincristine tumor treatment in fast contracting EDL muscle primarily reflects a decrease in myonuclear domain (Fig. 8). In contrast, we find that the deficient myofiber growth in SOL muscles is equally associated with a reduction of myonuclear number and domain after single or combined treatments. These damaging insults to the prepubescent myofibers are striking even weeks post-treatment and have not been described before now; however, to what extent these phenotypes persist throughout life is unknown. Clinical findings taken together with our data suggest that some normal tissue damage may be inevitable after pediatric cancer treatment despite optimal, localized therapeutic dosing and delivery. However, interventions such as physical rehabilitation and exercise offer hope for improvement of muscular decline experienced in childhood cancer survivors^79^. In the future, the preclinical pediatric cancer model described here will allow us to investigate long-term deficiencies consistent with premature sarcopenia in normal muscle tissue in order to understand how to retain youthful physiological function as long as possible in survivors of pediatric cancer.

## Methods

### Experimental animals statement

This study was carried out in strict accordance with the recommendations in the Guide for the Care and Use of Laboratory Animals of the National Institutes of Health. All procedures involving animals were approved by the Institutional Animal Care and Use Committee (IACUC) at the University of Rochester called the University Committee on Animal Resources (UCAR).

### RMS-luc-RFP cell line development

Embryonic rhabdomyosarcoma cell line M3-9-M was a kind donation from the Mackall lab at Stanford University^36^. Luciferase was inserted into a pCDH-CMV-MCS-EF1α-RFP + Puro cloning and expression lentivector (System Biosciences, CD516B-2). M3-9-M cells were infected with this lentiviral vector to yield stable expression of luciferase and RFP (RMS-luc-RFP). RFP was used for selection of positive clones. Cells were cultured in RPMI 1640 with l-glutamine (Gibco, 11875119) with added 10% heat‐inactivated fetal bovine serum (Gibco, 10082147), HEPES buffer (Gibco, 15,630,080), 1% MEM non‐essential amino acids (Gibco, 11140050), 1% sodium pyruvate (Gibco, 11360070), and 1% penicillin/streptomycin (Gibco, 15140122).

### Tumor Implantation

3–4-week-old male C57BL/6 J mice were housed in the animal facility with free access to standard rodent chow and water. Tumor implantation was performed the day after arrival in the vivarium. All mice were obtained from Jackson Laboratory (Bar Harbor, ME).

3-week-old C57BL/6 J mice were sedated with isoflurane. Fur was removed from the dorsal surface of the right hindlimb from the ankle to the hip. 100,000 RMS-luc-RFP tumor cells per mouse were resuspended in a solution of 1:1 matrigel (Corning #354234)/sterile PBS and injected in 30 µl into the outer lateral portion of the right gastrocnemius muscle with a standard insulin syringe, cooled on ice.

### In vivo imaging

RMS cell-injected mice were assessed daily following implantation, and tumor growth/recession was monitored using an IVIS spectrum in vivo imaging system (PerkinElmer). For these assays, mice were sedated with isoflurane, i.p. injected with approximately 100 µl of 30 mg/ml luciferin (150 mg/kg, d-Luciferin sodium salt, Gold Biotechnology, LUCNA-100), and imaged for bioluminescence detection after 15 min under sedation. RMS cell-injected mice were further assessed for bioluminescence using the IVIS directly following three/five days of radiation treatment (afternoon of the last treatment) and the beginning of the following week to assess tumor volume change, then weekly thereafter to monitor tumor growth/recurrence. The mice were then euthanized. Any mice with substantial tumor regrowth were euthanized at two weeks post-radiation treatment, or sooner if their condition declined.

### SARRP radiation

All radiation was delivered using the Small Animal Radiation Research Platform (SARRP, XStrahl) using a 10 × 10 mm collimator. Mice were anaesthetized with vaporized isoflurane during all radiation treatments; anesthesia was maintained throughout the radiation procedure via a nose cone. Using the customized single-mouse SARRP bed, the right foot of each mouse was taped with the bottom of the foot facing down with the leg slightly extended; the tail was positioned away from the right leg to minimize exposure of the genitalia. Fractionated radiation was administered to juvenile mice (~ 4 weeks old) at 5 days post RMS cell implantation using either 3 treatments of 8.2 Gy radiation every other day (Monday, Wednesday, Friday) or 5 treatments of 4.8 Gy (M-F) for the combined drug study. Localized delivery to the lower right limb (targeting the 10 × 10 mm area from the gastrocnemius-Achilles musculotendinous junction to just above the knee) was visualized with a pre-treatment computed tomography (CT) scan. The dosing isocenter was placed at the center of the tibia, offset to the center of the tumor bulk. The beam was angled to avoid major organs, principally the bladder and other pelvic organs. For each irradiation, a dose volume histogram (DVH) was generated to confirm full dose deposition in the tumor, generate isodose curves in the surrounding soft tissue and bone and confirm negligible exposure to critical organs (e.g., bladder).

### Vincristine treatment

Vincristine sulfate (Sigma-Aldrich, V8388) was dissolved in sterile water at 100X or 20 mg/mL concentration and kept at − 20 °C. This stock was then freshly diluted at 1:100 in saline (0.9% Sodium Chloride Irrigation, Baxter, 2F7123) to 0.2 mg/mL and adjusted to 1 mg/kg mouse body weight for each mouse (5 µl per gram)^80^. 3.5–4-week-old C57Bl/6 J mice were injected i.p. with vincristine or vehicle (saline only) using a standard insulin syringe 3 days following RMS cell implantation (two days prior to first radiation treatment). It was essential that mouse body weight be approximately 12 g or more at the time of vincristine injection to avoid morbidity and mortality associated with the toxicity of the drug.

### Rotarod test

Prior to the experiment, for 3 consecutive days mice were trained for 15 min at 15 rpm on an Economex rotarod with a 3 cm diameter rod (Columbus Instruments). On the fourth day, mice underwent the endurance fatigue testing. Rotation speed began at 14 rpm, followed by an increase in speed by 1 rpm every 5 min up to 23 rpm; then a maximum speed of 23 rpm was used for the final 15 min of the 1 h protocol. In the event of a fall, the fall was recorded and the mouse returned to the rod. The number of cumulative falls, binned every 5 min, was determined for each animal. Pass/fail cut-off of 7 falls was determined based on the results of the Control group^49^.

### Immunofluorescence

Immunofluorescent microscopy was conducted as described in detail elsewhere^30,48,52,68,81^. Briefly, dissected gastrocnemius muscles containing RMS tumors (previously fixed in 4% PFA) or fresh EDL/SOL muscles were washed in PBS and incubated overnight at 4 °C in 30% sucrose, flash frozen in OCT, cryosectioned at 10 μm, and stored at − 80 °C prior to staining. Muscle sections were fixed for 3 min in 4% paraformaldehyde (PFA). Tissue sections were permeabilized with PBS-T (0.2% Triton X-100) for 10 min and blocked in 10% Normal Goat Serum (NGS; Jackson Immuno Research) in PBS-T for 30 min at room temperature. When mouse primary antibodies were used, sections were additionally blocked in 3% AffiniPure Fab fragment goat anti-mouse IgG(H + L) (Jackson Immuno Research) with 2% NGS in PBS at room temperature for 1 h. Primary antibodies were incubated in 2% NGS/PBS at 4 °C overnight and with secondary antibodies for 1 h at RT. DAPI staining was performed to identify nuclei. All slides were mounted with Fluoromount-G (SouthernBiotech).

### Antibodies

The following antibodies were used as described in detail elsewhere^30,48,52,68,81^: Mouse anti-MyoD (1:250, BD Biosciences #554130), rabbit anti-Myogenin (1:250, Abcam, ab124800), rat anti-Laminin (1:1000, Sigma-Aldrich, L0663), mouse anti-Pax7 (1:100, Developmental Studies Hybridoma Bank), BA-D5 (MyHC-I, mouse IgG2b, 1:40, DSHB), SC-71 (MyHC-IIA, mouse IgG1, 1:40, DSHB), BF-F3 (MyHC-IIB, mouse IgM, 1:40, DSHB), AlexaFluor 488-conjugated goat anti-mouse IgG (1:1500, Life Technologies, A-11001), AlexaFluor 488-conjugated goat anti-rabbit IgG (1:1500, Life Technologies, A-11034), AlexaFluor 647-conjugated goat anti-rat IgG (1:1500, Life Technologies, A-21247), Alexa Fluor 405-conjugated goat anti-mouse IgG2b (1:1500, Thermo Fisher Scientific, A-21141), Alexa Fluor 488-conjugated goat anti-mouse IgM (1:1500, Thermo Fisher Scientific, A-21042), Alexa Fluor 594-conjugated goat anti-mouse IgG1 (1:1500, Thermo Fisher Scientific, A-21125).

### Fixed single fiber analysis

For single myofiber size and myonuclear analysis, whole limbs were fixed in 4% PFA for 48 h prior to muscle dissection. Fixed muscles were then dissociated by incubation in 40% NaOH for 2 h^30,35,68^. Single myofibers were gently titrated and washed in PBS prior to staining with DAPI. Myofiber CSA was determined using Image J software. The diameter of the myofiber was measured at three points along the fiber and average to get cross-sectional area (CSA). Myonuclear number was calculated per millimeter length (MN/mm) in the center portion of the myofiber. Myonuclear domain was calculated by dividing myofiber volume [myofiber length (per mm) multiplied by average CSA] by number of myonuclei (per mm). All imaging and quantification were performed blinded. We assessed ~ 1.5 mm length regions from ~ 50 individual myofibers per n.

### Data analysis

Immunofluorescent images were analyzed using ImageJ software as described in detail elsewhere^30,48,52,68,81^. Results are presented as mean + SEM. Statistical significance was determined by Student’s *t*-tests for simple comparison or by one-way ANOVA and Bonferroni multiple comparisons test for multiple comparisons with Graph Pad Prism software. P < 0.05 was considered statistically significant.

## Supplementary information

Supplementary Information

## Data Availability

The datasets used and/or analyzed during the current study are available from the corresponding author on reasonable request.
