# Supplementary material for: Chemoradiation impairs myofiber hypertrophic growth in a pediatric tumor model
Source: Sci Rep. 2020 Nov 11;10:19501. doi: 10.1038/s41598-020-75913-w (PMC7659015; doi:10.1038/s41598-020-75913-w)
Supplement: Supplementary file 1 — Supplementary Information [file 41598_2020_75913_MOESM1_ESM.pdf]

Chemoradiation impairs myofiber hypertrophic growth in a pediatric tumor model.

Supplementary Information

Authors:

Paris D. Nicole, Kallenbach G. Jacob, Bachman F. John, Blanc S. Roméo, Johnston J. Car, Hernady Eric, Williams P. Jacqueline, and Chakkalakal V. Joe

A

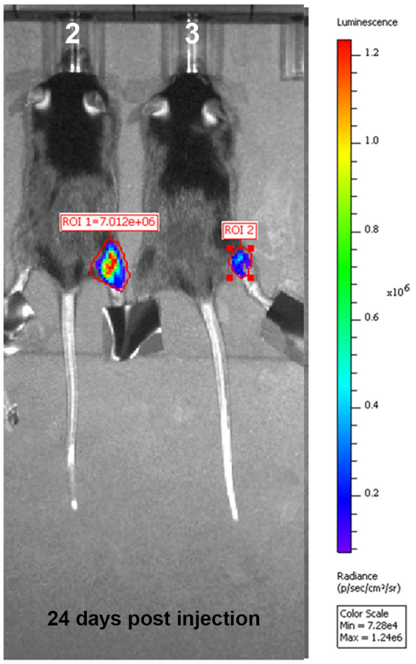

B

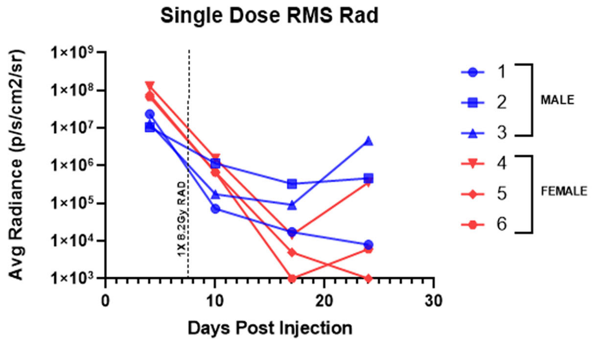

C

Single Dose Rad Final Tumor Size	
Male 1	0
Male 2	3 x 1.9cm
Male 3	1 x 1.8cm
Female 1	0.8 x 0.7cm
Female 2	0
Female 3	0

Supplementary Fig. 1. Single dose of 8.2 Gy radiation is not sufficient to eliminate RMS tumors. IVIS luminescent detection **A**) merged with brightfield image and **B**) quantified as average radiance (p/s/cm^2/sr) recorded weekly for 4 weeks post tumor cell implantation. **C**) Quantification of tumor size at time of harvest 3 weeks post radiation treatment. N = 3 mice per sex.

A

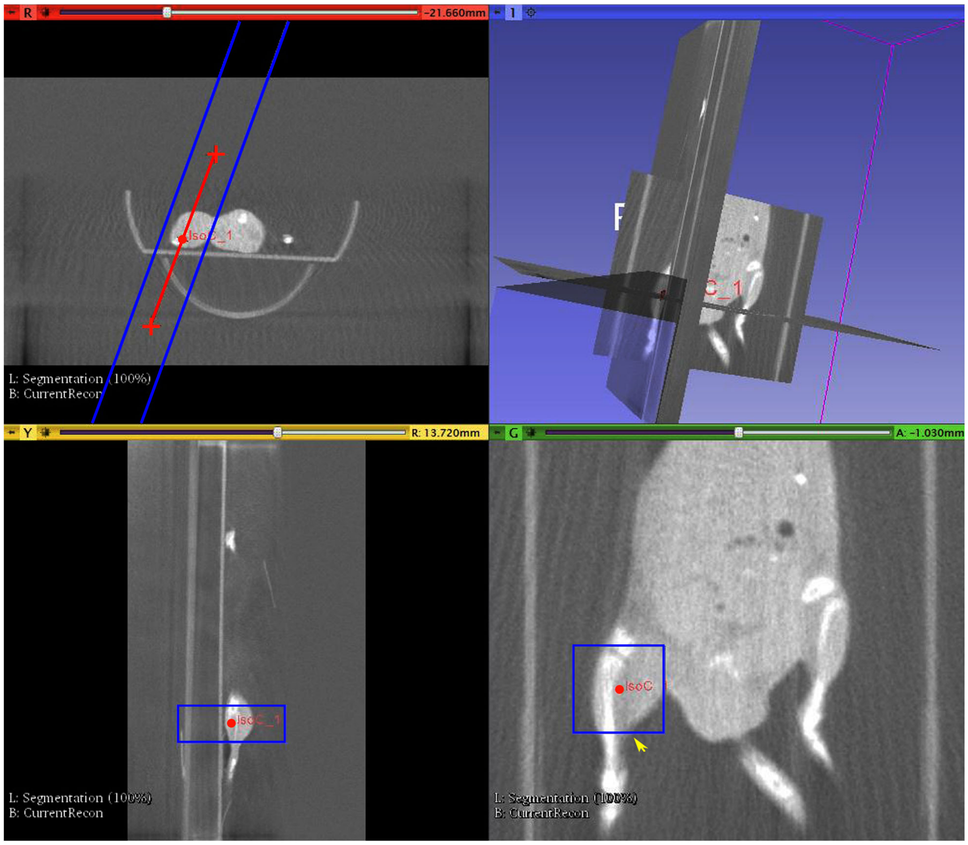

B

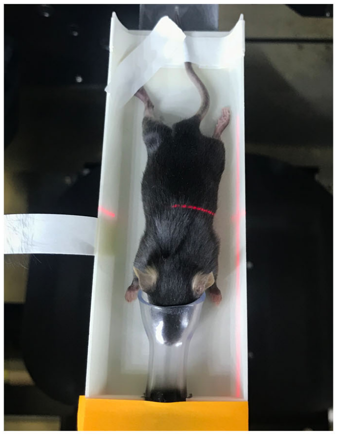

Supplementary Fig. 2. Placement of SARRP Radiation Field.
A) Screenshot of MuriPlan software showing CT image from four viewpoints. Blue lines outline the radiation field, red circles label the isocenter. Yellow arrow indicates tapering of gastrocnemius used as a landmark for placement of the radiation field. **B**) Placement of sedated mouse on radiation bed of SARRP. Note that the taping of the right foot in this orientation is necessary for a clear and accurate CT image.

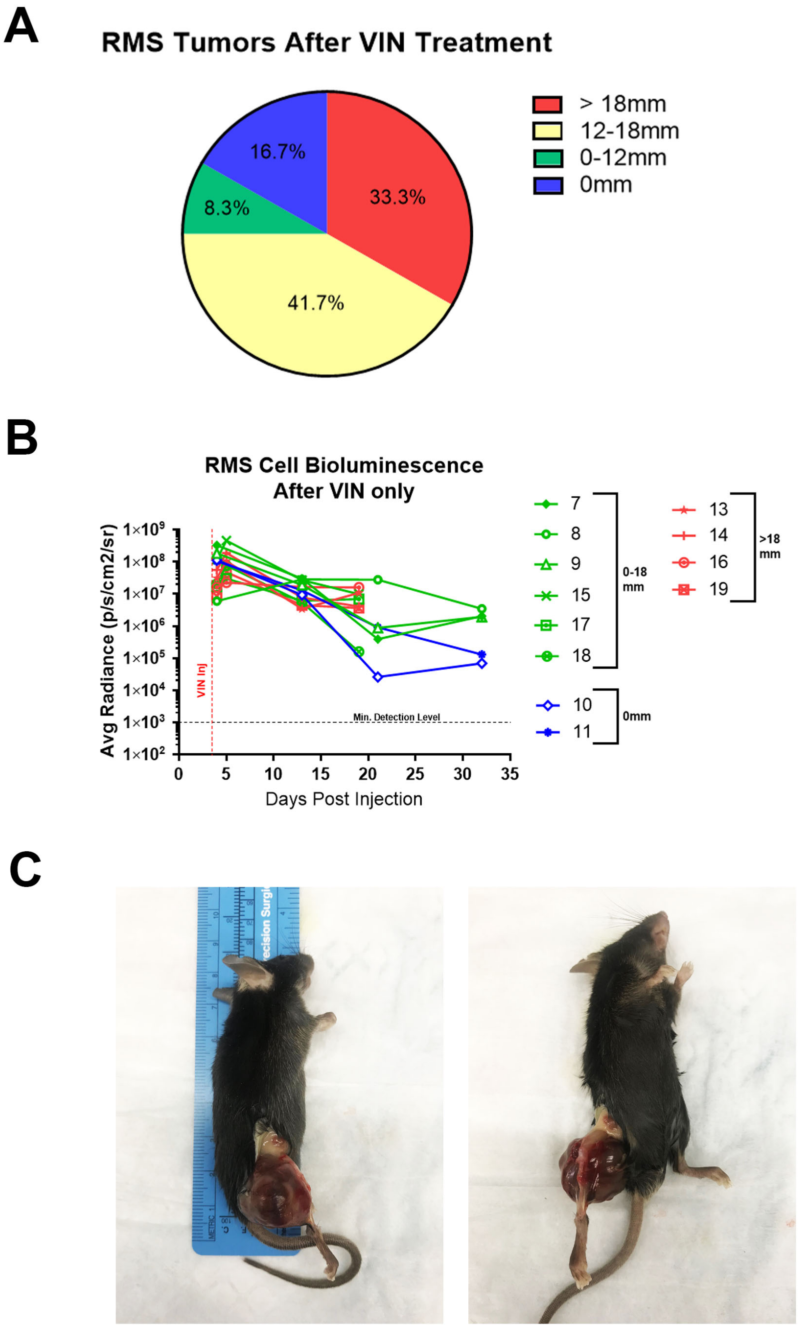

Supplemental Fig. 3. RMS tumor response to Vincristine treatment. **A**) Chart representing the percent of all tumors treated with Vincristine only with the corresponding size at time of harvest, at 8 weeks old approximately 4 weeks following treatment. N = 12 mice. **B**) IVIS bioluminescent detection quantified as average radiance (p/s/cm^2/sr) recorded for 5 weeks post tumor cell implantation. Graph represents only Vincristine treated RMS tumor bioluminescence and is further color-coded by size of tumor at time of harvest. **C**) Representative images of a tumor >18mm in size, from the side (left) and the top (right).

A EDL Statistics in detail

Cross-Sectional Area (CSA)		
Source of Variation	P value	
Interaction	0.0267	*
Radiation	<0.0001	****
Chemotherapy	0.0002	***
Tukey's Test	**P value**	
CL:VIN vs CL:Veh	ns	0.1222
CL:VIN vs. RL:VIN	***	0.0001
CL:VIN vs. RL:Veh	ns	0.2631
CL:Veh vs. RL:VIN	****	<0.0001
CL:Veh vs. RL:Veh	**	0.0079
RL:VIN vs. RL:Veh	***	0.0009

Myonuclear Number (MN)		
Source of Variation	P value	
Interaction	0.8177	ns
Radiation	0.0013	**
Chemotherapy	0.7726	ns
Tukey's Test	**P value**	
CL:VIN vs CL:Veh	ns	>0.9999
CL:VIN vs. RL:VIN	*	0.0287
CL:VIN vs. RL:Veh	*	0.0488
CL:Veh vs. RL:VIN	*	0.0271
CL:Veh vs. RL:Veh	*	0.0459
RL:VIN vs. RL:Veh	ns	0.98

Myonuclear Domain (MD)		
Source of Variation	P value	
Interaction	0.009	**
Radiation	<0.0001	****
Chemotherapy	<0.0001	****
Tukey's Test	**P value**	
CL:VIN vs CL:Veh	*	0.0258
CL:VIN vs. RL:VIN	***	0.0002
CL:VIN vs. RL:Veh	ns	0.9915
CL:Veh vs. RL:VIN	****	<0.0001
CL:Veh vs. RL:Veh	*	0.0382
RL:VIN vs. RL:Veh	***	0.0001

Fiber Volume		
Source of Variation	P value	
Interaction	0.1914	ns
Radiation	<0.0001	****
Chemotherapy	<0.0001	****
Tukey's Test	**P value**	
CL:VIN vs CL:Veh	*	0.016
CL:VIN vs. RL:VIN	***	0.0002
CL:VIN vs. RL:Veh	ns	0.2639
CL:Veh vs. RL:VIN	****	<0.0001
CL:Veh vs. RL:Veh	**	0.0014
RL:VIN vs. RL:Veh	**	0.0014

B SOL Statistics in detail

Cross-Sectional Area (CSA)		
Source of Variation	P value	
Interaction	0.0011	**
Radiation	<0.0001	****
Chemotherapy	<0.0001	****
Tukey's Test	**P value**	
CL:VIN vs CL:Veh	****	<0.0001
CL:VIN vs. RL:VIN	**	0.0091
CL:VIN vs. RL:Veh	ns	0.7824
CL:Veh vs. RL:VIN	****	<0.0001
CL:Veh vs. RL:Veh	****	<0.0001
RL:VIN vs. RL:Veh	*	0.0323

Myonuclear Number (MN)		
Source of Variation	P value	
Interaction	0.2917	ns
Radiation	0.0038	**
Chemotherapy	0.0029	**
Tukey's Test	**P value**	
CL:VIN vs CL:Veh	*	0.0221
CL:VIN vs. RL:VIN	ns	0.2461
CL:VIN vs. RL:Veh	ns	0.9989
CL:Veh vs. RL:VIN	**	0.0017
CL:Veh vs. RL:Veh	*	0.0268
RL:VIN vs. RL:Veh	ns	0.2042

Myonuclear Domain (MD)		
Source of Variation	P value	
Interaction	0.0431	*
Radiation	0.0008	***
Chemotherapy	0.0058	**
Tukey's Test	**P value**	
CL:VIN vs CL:Veh	*	0.0107
CL:VIN vs. RL:VIN	ns	0.2543
CL:VIN vs. RL:Veh	ns	0.706
CL:Veh vs. RL:VIN	***	0.001
CL:Veh vs. RL:Veh	**	0.0028
RL:VIN vs. RL:Veh	ns	0.7847

Fiber Volume		
Source of Variation	P value	
Interaction	0.1013	ns
Radiation	<0.0001	****
Chemotherapy	0.0011	**
Tukey's Test	**P value**	
CL:VIN vs CL:Veh	**	0.0056
CL:VIN vs. RL:VIN	*	0.0209
CL:VIN vs. RL:Veh	ns	0.4275
CL:Veh vs. RL:VIN	***	0.0001
CL:Veh vs. RL:Veh	***	0.0009
RL:VIN vs. RL:Veh	ns	0.1958

Supplemental Fig. 4. Detailed statistical analyses of myofiber characteristics. A) EDL myofiber assay detailed statistics from Two-way ANOVA with Tukey's multiple comparisons test. **B**) SOL myofiber assay detailed statistics from Two-way ANOVA with Tukey's multiple comparisons test. CL = Contralateral Leg, RL = Irradiated Leg, Veh = Vehicle, VIN = Vincristine.

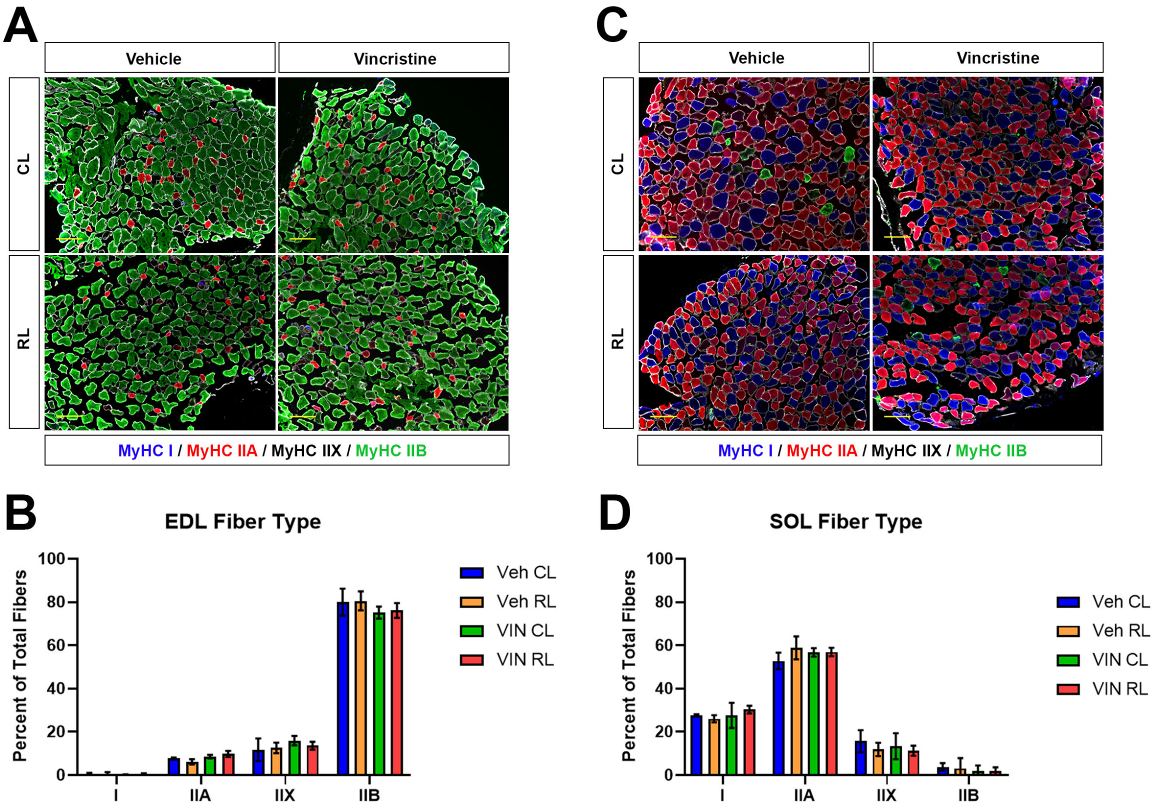

Supplementary Fig 5. Chemoradiation does not cause significant muscle fiber type changes 3 weeks post treatment. Representative MyHC IIa (red), MyHC I (blue), MyHC IIX (black), and MyHC IIB (green) immunofluorescent images and quantification of cross-sections of **A, B**) EDL and **C, D**) SOL. Scale = 100µm.
CL = Contralateral Leg, RL = Irradiated Leg. N = 3 mice/group
